# The impact of timing of temozolomide chemoradiotherapy for newly diagnosed glioblastoma on patient overall survival: A multicenter retrospective study

**DOI:** 10.1093/noajnl/vdae194

**Published:** 2024-11-12

**Authors:** Arthur C K Lau, Brandon L H Chan, Carly S K Yeung, Lai-Fung Li, Danny T M Chan, Michael W Y Lee, Tony K T Chan, Jason M K Ho, Ka-Man Cheung, Teresa P K Tse, Sarah S N Lau, Joyce S W Chow, Natalie M W Ko, Herbert H F Loong, Aya El-Helali, Wai-Sang Poon, Peter Y M Woo

**Affiliations:** Department of Neurosurgery, Prince of Wales Hospital, Hong Kong; Department of Neurosurgery, Prince of Wales Hospital, Hong Kong; Department of Neurosurgery, Prince of Wales Hospital, Hong Kong; Division of Neurosurgery, Department of Surgery, Queen Mary Hospital, Hong Kong; Department of Neurosurgery, Prince of Wales Hospital, Hong Kong; Department of Neurosurgery, Pamela Youde Nethersole Eastern Hospital, Hong Kong; Department of Neurosurgery, Princess Margaret Hospital, Hong Kong; Department of Neurosurgery, Tuen Mun Hospital, Hong Kong; Department of Clinical Oncology, Queen Elizabeth Hospital, Hong Kong; Department of Neurosurgery, Princess Margaret Hospital, Hong Kong; Division of Neurosurgery, Department of Surgery, Queen Mary Hospital, Hong Kong; Department of Neurosurgery, Queen Elizabeth Hospital, Hong Kong; Department of Neurosurgery, Kwong Wah Hospital, Hong Kong; Department of Clinical Oncology, The Chinese University of Hong Kong, Hong Kong; Department of Clinical Oncology, The University of Hong Kong, Hong Kong; Department of Neurosurgery, Prince of Wales Hospital, Hong Kong; Department of Neurosurgery, Prince of Wales Hospital, Hong Kong

**Keywords:** chemoradiotherapy, extent of resection, glioblastoma, methylguanine-methyltransferase promoter methylation, overall survival, temozolomide

## Abstract

**Background:**

The optimal timing of initiating adjuvant temozolomide (TMZ) chemoradiotherapy after surgery in patients with glioblastoma is contentious. This study aimed to determine whether the timing of adjuvant treatment affects their overall survival (OS).

**Methods:**

Consecutive adult patients with histologically-confirmed newly diagnosed glioblastoma treated with adjuvant TMZ chemoradiotherapy across all neurosurgical centers in Hong Kong between 2006 and 2020 were analyzed. The surgery-to-chemoradiotherapy (S-CRT) interval was defined as the date of the first surgery to the date of initiation of adjuvant TMZ chemoradiotherapy.

**Results:**

Four hundred and forty-one patients were reviewed. The median S-CRT interval was 40 days (interquartile range [IQR]: 33–47) and the median overall survival (mOS) was 16.7 months (95% CI: 15.9–18.2). The median age was 58 years (IQR: 50–63). Multivariable Cox regression with restricted cubic splines identified a nonlinear relationship between the S-CRT interval and mOS. *Post hoc* analysis-derived S-CRT intervals revealed that early CRT (<5 weeks; adjusted hazard ratio [aHR]: 1.11; 95% CI 0.90–1.37) or late CRT (>9–12 weeks; aHR 1.07; 95% CI 0.67–1.71) were not significantly associated with OS. Subgroup analyses for the extent of resection (EOR) and p*MGMT* methylation status revealed no significant difference in treatment timing on OS.

**Conclusion:**

The timing of adjuvant TMZ chemoradiotherapy, if commenced within 12 weeks after glioblastoma diagnosis, did not influence OS regardless of EOR or p*MGMT* methylation status. Clinical judgment should be exercised in optimizing the timing of initiating adjuvant therapy.

## Lay Summary

Glioblastoma is an aggressive brain cancer. The treatment for glioblastoma includes surgery followed by a combination of radiotherapy and a chemotherapeutic agent called temozolomide (TMZ). However, the best time to start this treatment after surgery is unclear. The authors of this study wanted to find out if the timing of this combined therapy after surgery affects how long patients live. To do this, they looked at the medical records of 441 patients with glioblastoma. Their results showed that patients who started combined TMZ and radiotherapy within 5 weeks of surgery lived about the same time as those who started 9–12 weeks after surgery.

**Key Points**

- Commencing chemoradiotherapy (CRT) within 3 months of surgery does not influence overall survival (OS).
- There is no OS benefit with earlier CRT, that is <5 weeks compared with later CRT, that is 9–12 weeks from diagnosis.
- Glioblastoma *MGMT* promoter methylation and repeat resection of recurrent tumors were predictors for OS.

**Importance of the Study**

Glioblastoma is the most common primary malignant brain tumor in adults and carries a poor prognosis. While the timely initiation of adjuvant temozolomide chemoradiotherapy (CRT) is often considered beneficial given the aggressive nature of these tumors, there are concerns over premature treatment, which has been linked to poorer overall survival (OS). We aimed to identify whether there is an optimal timing of adjuvant CRT by considering important factors including extent of resection and O^6^-methylguanine-DNA methyltransferase promoter methylation (p*MGMT*) status. By modeling the surgery-to-CRT interval (S-CRT) as a continuous variable with restricted cubic splines, we identified an optimal period of S-CRT between 5 and 9 weeks post-surgery. Subsequent multivariable analysis did not detect significant associations between S-CRT durations, within 3 months of diagnosis, and OS. Clinical judgment remains essential in determining the timing of adjuvant CRT for glioblastoma patients.

Glioblastoma is the most common primary malignant brain tumor in adults and carries a poor prognosis.^1^ Multimodal therapy is the standard of care for patients and comprises maximal safe resection followed by adjuvant temozolomide (TMZ) chemoradiotherapy (CRT).^2,3^ The standard treatment dose for TMZ chemotherapy is 75 mg/m^2^/day for 6 weeks and is prescribed concomitantly with radiotherapy of 60 Gy over 30 fractions.^2,3^ Subsequent maintenance chemotherapy comprises TMZ 150–200 mg/m^2^/day for 5 days every 4 weeks for 6 cycles.^4,5^ However, despite such treatment the 2-year survival rate is only 18% and median overall survival (mOS) ranges from 10 to 14 months.^4,5^ Prognostic factors include age, Karnofsky performance status (KPS), O^6^-methylguanine-DNA methyltransferase promoter (p*MGMT*) methylation status, and extent of resection (EOR).^6–9^ Numerous efforts have been made to investigate whether adjustments to adjuvant oncologic therapy could improve OS. In particular, with regard to dose-dense metronomic TMZ administration, alternate weekly administration, or extended treatment beyond 6 cycles, numerous randomized-controlled trials did not demonstrate a clear benefit with these adjustments.^10–13^

Relatively few investigators have assessed whether the time interval between surgery and the initiation of adjuvant treatment influences OS in the TMZ era. Given the aggressive nature of glioblastoma, timely adjuvant treatment may improve survival, but studies have noted that premature adjuvant treatment could be detrimental.^14–20^ There remains no consensus on the optimal timing of commencing adjuvant TMZ CRT. Previous studies were limited by highly heterogeneous patient populations, pooling together patients treated with CRT, and those treated with radiotherapy alone.^15,20,21^ Others did not account for important prognosticators, particularly p*MGMT* methylation status, or EOR.^16,19,22^ The purpose of this study was to examine whether the timing of adjuvant TMZ CRT is associated with survival in patients with newly diagnosed glioblastoma by comprehensively reviewing these important confounding factors.

## Materials and Methods

This was a multi-centre retrospective cohort study of consecutive adult patients (≥18 years) with histologically-confirmed newly diagnosed glioblastoma in Hong Kong. The diagnosis of glioblastoma was determined in accordance with the World Health Organization (WHO) Classification of Central Nervous System Tumors, fourth edition.^23^ This study was approved by the Institutional Review Board of the Hospital Authority (HA) of Hong Kong (reference number: KC/KE-18-0262/ER-4). Universal healthcare is offered in Hong Kong and is delivered by the HA, a highly-subsidized government statutory body that offers care for more than 90% of inpatient bed days in the city.

### Patient Population

**Figure 1.**
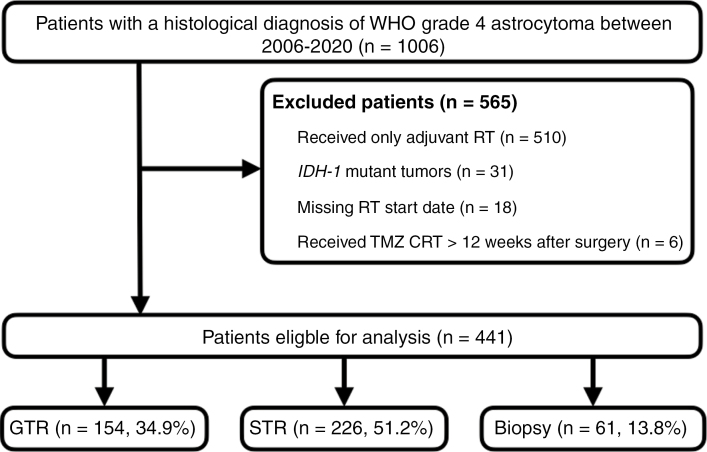
Patient cohort inclusion and exclusion characteristics. Abbreviations: CRT, chemoradiotherapy; GTR, gross total resection; RT, radiotherapy; STR, subtotal resection; TMZ, temozolomide.

Data was reviewed for adult patients with newly diagnosed glioblastoma who received adjuvant TMZ CRT at all of Hong Kong’s 7 neurosurgical units between 1 January 2006 and 31 April 2020 from the Hong Kong Glioblastoma Registry (HK-GBM Registry).^5^ The registry is a centralized repository of prospectively collected information of consecutive patients with glioblastoma treated by Hong Kong’s public healthcare system.^5^ Patients that had *IDH-1* mutant tumors, received TMZ or another chemotherapeutic agent for a preexisting lower grade glioma, received radiotherapy (RT) alone, started RT > 12 weeks after diagnosis, or were unable to complete the entire course of concomitant TMZ CRT were excluded (Figure 1).

Patient-related data, including age, gender, and preoperative KPS were documented. Tumor-related data including tumor location and p*MGMT* methylation status were collected. Treatment-related factors such as EOR, date of first operation, and date of commencement of adjuvant TMZ CRT were also retrieved. The use of regional treatments such as interstitial chemotherapy, laser interstitial thermal therapy, intracavitary RT, or tumor-treating fields were collected. In addition, second-line therapy upon tumor recurrence such as re-irradiation, systemic treatment such as lomustine, immunotherapy or bevacizumab, and repeat resection were documented. Extent of resection was determined either by reviewing postoperative day-1 magnetic resonance imaging (MRI) gadolinium contrast-enhanced scans on workstations installed with Centricity Enterprise Web (General Electric Medical Systems) image viewers or when such scans were not available, by the neurosurgeon’s assessment documented in the operation records. Extent of resection was broadly categorized into either gross total resection (GTR), subtotal resection (STR), or biopsy in accordance with the Response Assessment in Neuro-oncology (RANO) criteria.^24^ All scans underwent centralized review by 2 clinicians (A.C.K.L. and B.L.H.C.), that had 12 months of neuro-oncology radiology experience, with regard to determining the imaging features of the tumor, assessing EOR, and detecting subsequent disease progression. When discrepancies arose between the scan reviewers, final arbitration was performed by the senior author (P.Y.M.W.). The surgery-to-chemoradiotherapy (S-CRT) interval was defined as the duration from the date of first surgery to the date of starting TMZ CRT. The primary endpoint of the study was OS as defined from the date of first surgery until death. Cases were censored by 30 June 2023.

### Statistical Analysis

To explore the impact of the timing of adjuvant oncologic therapy on OS, the S-CRT interval was first subject to univariable Cox proportional hazards analysis. An *a priori* approach was first adopted where the 12-week S-CRT duration was analyzed as a categorical variable and stratified into either 4- or 6-week time intervals representing either early or late adjuvant treatment. For the former categorization, S-CRT duration was divided into <4 weeks (“early”), ≥4–8 weeks (“reference”), and >8–12 weeks (“late”). The latter categorization was dichotomized to ≤6 weeks (“reference”) and >6–12 weeks (“late”). Covariates included in the multivariable Cox regression model were age, gender, preoperative KPS, and p*MGMT* methylation status. Adjusted hazard ratios (aHR) were determined by multivariable Cox regression. Survival probabilities were evaluated by Kaplan-Meier analysis and log-rank testing. Subsequent *post hoc* analysis was performed by first analyzing the S-CRT duration as a continuous variable that was subsequently modeled by a restricted cubic spline (RCS) function with 5 knots. The median S-CRT interval was set as the reference with an adjusted HR of 1 and 3 interval categories were then established. Since the RCS function does not assume linearity, this approach was utilized to explore the effects of the S-CRT interval on OS.^15,25^ The number of knots was chosen to minimize the Akaike Information Criteria (AIC), which ensured goodness of fit while avoiding over-fitting of the data.^26^ Sensitivity analyses were carried out in which interaction terms were introduced to the multivariable Cox model to test for interaction between covariates and the S-CRT interval.

Differences in baseline patient characteristics between early, late, and reference groups were compared utilizing Pearson’s chi-square testing, and one-way analysis of variance (ANOVA). Subgroup analyses were conducted based on EOR and p*MGMT* methylation status. A *P*-value of <.05 was considered statistically significant. All tests were performed utilizing R (version 4.2.0, R Foundation) or the Statistical Package for the Social Sciences software version 21.0 (SPSS Inc.).

## Results

### Patient, Tumor, and Treatment Characteristics

A total of 1006 adult patients with histologically-proven newly diagnosed WHO grade 4 astrocytoma were identified during the study period of which 441 (43.8%) glioblastoma patients were eligible for review. The follow-up rate was 90.9% and the median follow-up duration was 87.4 months (IQR: 57.6–136.7). The median age was 58 years (IQR 50–63) (mean age: 54.8 ± 12.3 years) and the female to male ratio was 1:1.6. Two hundred nine (47.4%) patients had a preoperative KPS of ≥80 and a third of tumors were located in the frontal lobe (32.2%, 142/441) (Table 1). Half of the patient cohort (47.3%, 165/441) had p*MGMT*-methylated tumors. One hundred fifty-four patients (34.9%) underwent gross total resection (GTR) and all completed standard-of-care concomitant TMZ CRT. The median S-CRT interval was 40 days (IQR: 33–47 days). None of the patients received regional therapy such as interstitial chemotherapy, intracavitary RT, laser interstitial thermal therapy, or tumor-treating fields as first-line treatment. None of the patients were recruited in a clinical intervention trial. As for second-line treatment, a quarter of patients (24.5%, 108/441) underwent repeat resection for first tumor recurrence followed by rechallenge TMZ (24.6%, 104/441) (Table 1).

**Table 1.** Baseline Patient, Tumor, and Treatment Characteristics

		*Post hoc* analysis-derived S-CRT intervals			
	Entire cohort *n* = 441 (%)	< 5 weeks (early) *n* = 155 (%)	5–9 weeks (reference) *n* = 267 (%)	>9–12 weeks (late) *n* = 19 (%)	*P*-value
**Patient factors**					
Male	272 (61.7)	89 (57.4)	175 (65.5)	8 (42.1)	NS
Age, y, median [IQR]	58.0 [50.0, 63.0]	58.0 [51.0, 63.0]	57.0 [48.0, 64.0]	60.0 [55.5, 65.0]	NS
Age > 70 years	32 (7.3)	13 (4.9)	18 (11.6)	1 (5.3)	NS
KPS ≥ 80	209 (47.4)	76 (49.0)	125 (46.8)	8 (42.1)	NS
**Tumor factors**					
Location					NS
Frontal lobe	142 (32.2)	48 (31.0)	88 (33.0)	6 (31.6)	
Temporal lobe	128 (29.0)	39 (25.2)	83 (31.1)	6 (31.6)	
Parietal lobe	113 (25.6)	43 (27.7)	65 (24.3)	5 (26.3)	
Occipital lobe	20 (4.5)	8 (5.2)	11 (4.1)	1 (5.3)	
Central core^a^	20 (4.5)	11 (7.1)	8 (3.0)	1 (5.3)	
Corpus callosum	10 (2.3)	3 (1.9)	7 (2.6)	0 (0)	
Cerebellum	8 (1.8)	3 (1.9)	5 (1.9)	0 (0)	
Tumor laterality					
Right	187 (44.6)	51 (34.9)	126 (49.6)	10 (52.6)	NS
p*MGMT* status					
Methylated	165 (47.3)	51 (43.6)	104 (48.1)	10 (62.5)	NS
**Treatment factors**					
Extent of resection					NS
GTR	154 (34.9)	57 (36.8)	89 (33.3)	8 (42.1)	
STR	226 (51.2)	80 (51.6)	138 (51.7)	8 (42.1)	
Biopsy	61 (13.8)	18 (11.6)	40 (15.0)	3 (15.8)	
**Second-line treatment**					
Repeat resection	108 (24.5)	37 (23.9)	66 (24.7)	5 (26.3)	NS
Re-irradiation	34 (7.7)	13 (8.4)	20 (7.5)	1 (5.3)	NS
Rechallenge temozolomide	104 (23.6)	37 (23.9)	61 (22.8)	6 (31.6)	NS
Lomustine	87 (19.7)	35 (22.6)	46 (17.2)	6 (31.6)	NS
NGS-guided targeted therapy	14 (3.2)	8 (5.2)	5 (1.9)	1 (5.3)	NS
Immunotherapy	3 (0.7)	1 (0.6)	2 (0.7)	0 (0)	NS
Tumor-treating fields	4 (0.9)	2 (1.3)	2 (0.7)	0 (0)	NS

Abbreviations: GTR, gross total resection; KPS, Karnofsky performance status; NGS, next-generation sequencing; p*MGMT*, methylguanine-methyltransferase promoter; S-CRT, surgery-to-chemoradiotherapy; STR, subtotal resection.

^a^Central core comprises of the insula, basal ganglia, and the thalamus.

### Overall Survival

The mOS was 16.7 months (95% CI: 15.9–18.2) for the entire cohort. The 12-month OS rate was 70.2% (307/ 441) and the 24-month OS rate was 34.4% (286/ 441). Multivariable Cox regression revealed that p*MGMT*-unmethylated glioblastoma was associated with a shorter mOS of 14.3 months compared to 24.7 months for patients with methylated tumors (aHR 1.89; 95% CI: 1.54–2.34) (Table 2). In addition, repeat resection was also noted to be an independent significant determinant for OS (aHR 0.65; 95% CI: 0.52–0.83). No other second-line therapy was observed to offer a survival benefit (*P*-value > .05).

**Table 2.** Univariable and Multivariable Cox Regression Analysis for Overall Survival

	Entire cohort *n* = 441 (%)	Univariable Cox regression	Multivariable Cox regression
		Hazard ratio (95% CI)	Adjusted hazard ratio (95% CI)
**Patient factors**			
Age > 70 y	32 (7.3)	1.19 (0.83–1.70)	
Male	272 (61.7)	1.04 (0.85–1.27)	
KPS < 80	232 (52.6)	1.19 (0.98–1.45)	
**Tumor factors**			
Location			
Frontal lobe	142 (32.2)	Ref	
Temporal lobe	128 (29.0)	1.01 (0.79–1.30)	
Parietal lobe	113 (25.6)	1.08 (0.83–1.40)	
Occipital lobe	20 (4.5)	1.27 (0.78–2.09)	
Central core	20 (4.5)	2.06 (1.27–3.34)	
Corpus callosum	10 (2.3)	1.61 (0.84–3.06)	
Cerebellum	8 (1.8)	0.84 (0.39–1.80)	
Left-sided tumor	232 (55.4)	1.15 (0.94–1.41)	
p*MGMT*-unmethylated	184 (52.7)	1.93 (1.54–2.43)	1.89 (1.54–2.34)
**Treatment factors**			
S-CRT interval			
* *A priori			
4-weekly intervals > 4-8 weeks	328 (74.4)	Ref	
Early ≤ 4 weeks	72 (16.3)	1.19 (0.91–1.56)	
Late > 8–12 weeks	41 (9.3)	0.92 (0.66–1.28)	
6-weekly intervals			
Early ≤ 6 weeks	169 (38.3)	Ref	
Late > 6–12 weeks	272 (61.7)	0.88 (0.72–1.07)	
* * *Post hoc*			
5–9 weeks	267 (60.5)	Ref	
Early < 5 weeks	155 (35.1)	1.11 (0.90–1.37)	
Late > 9–12 weeks	19 (4.3)	1.07 (0.67–1.71)	
Extent of resection			
GTR	154 (34.9)	Ref	
STR	226 (51.2)	1.02 (0.82–1.27)	
Biopsy	61 (13.8)	1.24 (0.91–1.68)	
Repeat resection	108 (24.5)	0.68 (0.54–0.86)	0.65 (0.52–0.83)

Abbreviations: GTR, gross total resection; KPS, Karnofsky performance status; p*MGMT*, methylguanine-methyltransferase promoter; S-CRT, surgery-to-chemoradiotherapy; STR, subtotal resection.

### Defining “Early” versus “Late” Surgery-to-Chemoradiotherapy Duration Intervals and their Impact on Overall Survival

**Figure 2.**
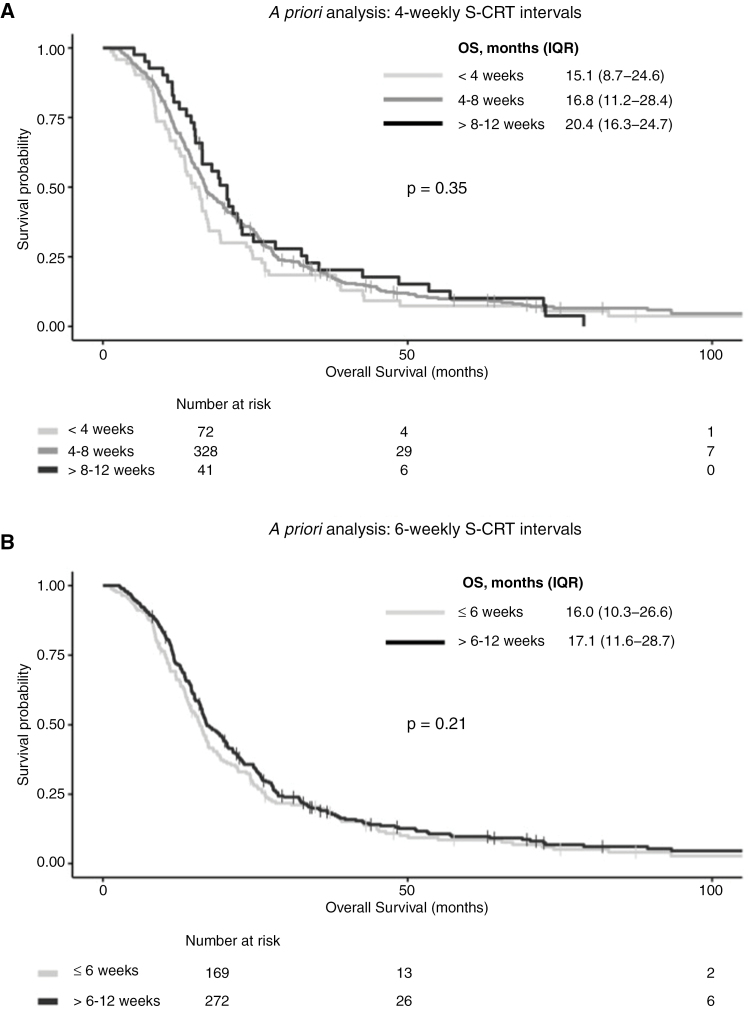
Kaplan-Meier survival analysis of a priori 4-weekly surgery-to-chemoradiotherapy (S-CRT) intervals (A) and 6-weekly C-SRT intervals (B).

**Figure 3.**
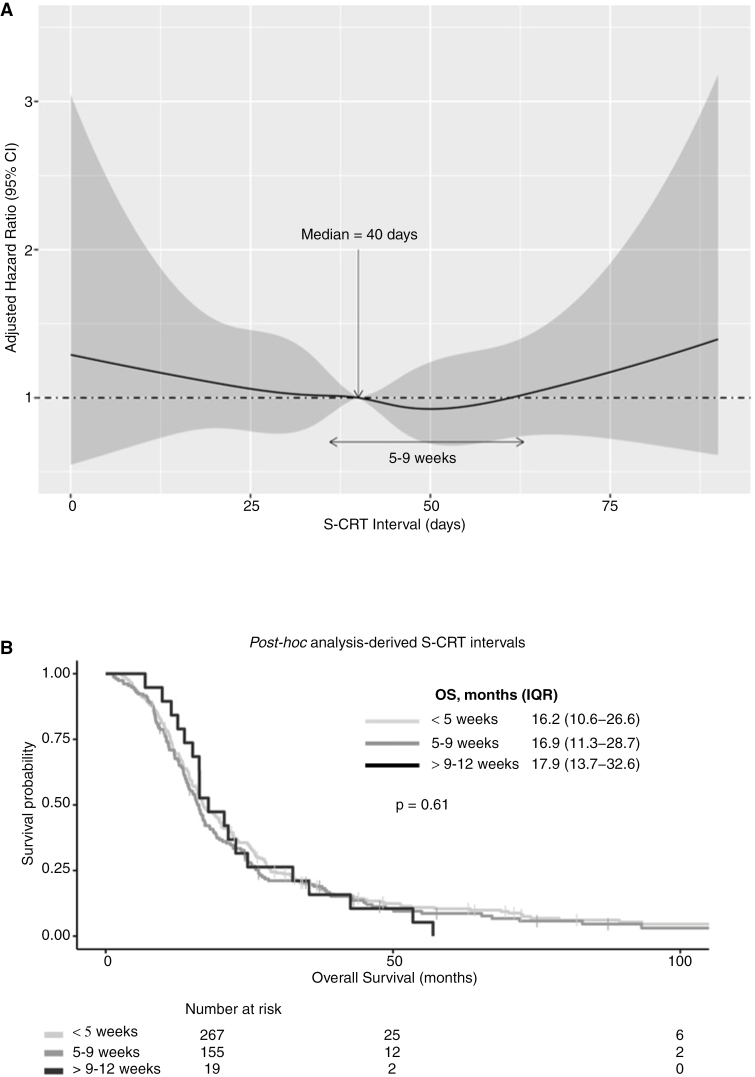
*Post hoc* analysis-derived surgery-to-chemoradiotherapy (S-CRT) intervals. S-CRT interval modeled as a continuous variable with restricted cubic splines (RCS), adjusted for gender, age, p*MGMT* methylation, KPS, and EOR (95% confidence intervals indicated by shaded area) (A). Kaplan-Meier survival curve of *post hoc* analysis-derived S-CRT intervals (B).

Kaplan-Meier survival analysis of *a priori* S-CRT categories of 4- or 6-weekly intervals did not reveal an optimal time period that conferred significantly longer OS (log-rank test, *P* > .05) (Figure 2). For *post hoc* analysis, the S-CRT interval was modeled as a continuous variable, and a nonlinear relationship with OS was observed (Figure 3A). When the median S-CRT interval of 40 days was used as a reference, the lowest adjusted HRs for OS were observed between weeks 5–9 (36–63 days). This suggested that when CRT was initiated during this time period after surgery, patients were more likely to have improved OS. Founded on this exploratory analysis, the S-CRT interval was categorized into 3 phases, early (<5 weeks), reference (5–9 weeks), and late (>9–12 weeks). Baseline patient and tumor characteristics were comparable between these S-CRT groups (Table 1). However, Kaplan-Meier analysis did not reveal a significant difference in OS (log-rank test, *P*: 0.61) (Figure 3B). Multivariable Cox regression, adjusted for patient-, tumor-related, and EOR factors, also showed no significant difference in OS between these 3 S-CRT interval categories (Table 2). Sensitivity analysis did not reveal any statistically significant interaction effects between S-CRT interval category, p*MGMT* methylation, and whether repeat resection was performed.

Subgroup analysis was performed to examine whether EOR had an impact on OS with regard to *post hoc* analysis-derived S-CRT interval categories. No significant difference in OS was observed between EOR and S-CRT intervals (Table 3). With regard to tumor p*MGMT* methylation status, no particular S-CRT interval demonstrated a significant impact on OS (Table 4).

**Table 3.** Extent of Resection Subgroup Analysis: Multivariable Cox Regression for Overall Survival

	GTR	STR	Biopsy
	Adjusted HR (95% CI)	Adjusted HR (95% CI)	Adjusted HR (95% CI)
**Patient factors**			
Age > 70 y	1.47 (0.81–2.65)	0.88 (0.50–1.55)	0.93 (0.24–3.58)
Male	1.28 (0.89–1.83)	0.97 (0.73–1.31)	0.92 (0.52–1.63)
KPS < 80	1.40 (0.97–2.01)	1.12 (0.84–1.50)	1.57 (0.82–3.01)
**Tumor factor**			
p*MGMT*-unmethylated	1.93 (1.25–2.99)	1.88 (1.36–2.61)	1.56 (0.75–3.28)
**Treatment factors**			
S-CRT interval			
5–9 weeks	Ref	Ref	Ref
Early < 5 weeks	1.02 (0.70–1.47)	1.13 (0.84–1.53)	1.40 (0.75–2.63)
Late > 9–12 weeks	1.67 (0.79–3.53)	1.20 (0.58–2.50)	0.46 (0.13–1.59)
Repeat resection	0.68 (0.44–1.05)	0.67 (0.47–0.94)	0.49 (0.27–0.90)

Abbreviations: GTR, gross total resection; HR, hazard ratio; KPS, Karnofsky performance status; p*MGMT*, methylguanine-methyltransferase promoter; S-CRT, surgery-to-chemoradiotherapy; STR, subtotal resection.

**Table 4.** Glioblastoma p*MGMT* Methylation Status Subgroup Analysis: Multivariable Cox Regression for Overall Survival

	p*MGMT*-unmethylated	p*MGMT*-methylated
	Adjusted HR (95% CI)	Adjusted HR (95% CI)
**Patient factors**		
Age > 70 y	1.01 (0.54–1.87)	1.10 (0.63–1.95)
Male	1.08 (0.79–1.49)	1.05 (0.75–1.48)
KPS < 80	1.46 (1.06–2.01)	1.09 (0.78–1.53)
**Treatment factors**		
S-CRT interval		
5–9 weeks	Ref	Ref
Early < 5 weeks	1.14 (0.82–1.58)	1.12 (0.78–1.61)
Late > 9–12 weeks	1.94 (0.83-4.51)	0.96 (0.49–1.88)
Extent of resection		
GTR	Ref	Ref
STR	0.97 (0.69–1.38)	1.16 (0.79–1.70)
Biopsy	1.00 (0.61–1.62)	1.72 (1.00-–2.93)
Repeat surgery	0.64 (0.45–0.92)	0.69 (0.47–1.03)

Abbreviations: GTR, gross total resection; HR, hazard ratio; KPS, Karnofsky performance status; p*MGMT*, methylguanine-methyltransferase promoter; S-CRT, surgery-to-chemoradiotherapy; STR, subtotal resection.

## Discussion

Despite 2 decades of investigating various adjuvant TMZ CRT regimens, EOR remains the only treatment-related predictor for OS. Currently, there is no evidence-based consensus on the most appropriate time for commencing CRT and previous studies have arrived at discordant conclusions.^14–22,27–32^ As a result, the latest European Association of Neuro-oncology (EANO) and Society for Neuro-oncology (SNO) clinical practice guidelines do not offer specific recommendations.^2,3^

The timing of CRT is often subject to a wide range of factors, including medical issues such as postoperative neurological recovery, the management of procedure-associated complications, and medical comorbidities. Logistical issues such as delays between referrals and consultations, particularly in resource-constrained institutions also exist. In Hong Kong, unlike other malignancies, there are no local clinical practice guidelines for when CRT should begin. Treatment timing is largely governed by the patient's postoperative KPS, scalp wound healing condition and EOR, whereby larger residual tumors are treated as a priority.^5^ It was noted that 35.1% of patients were able to start within 5 weeks of surgery and only 4.3% of patients commenced at 9–12 weeks. We observed that for S-CRT intervals of up to 12 weeks, OS was unaffected regardless of EOR and p*MGMT* methylation status. This implies that a 12-week window between surgery and CRT could be allowed for postoperative recovery, and for coordinating a treatment plan.

Our study findings are in contrast with those that deal with other malignancies. Earlier commencement of adjuvant RT was found to improve OS in head and neck cancer patients, with the National Comprehensive Cancer Network guidelines stipulating that RT should be initiated within 6 weeks after surgery.^33,34^ For breast cancer, the European Society of Medical Oncology guidelines recommend initiating systemic chemotherapy ideally within 4–6 weeks after diagnosis for early-stage disease.^35^ As for RT, the Japanese Breast Cancer Society advocates starting within 20 weeks of surgery and the American Society for Radiation Oncology recommends treatment as soon as within 2 weeks for early-stage invasive breast cancer.^36,37^ It was therefore postulated that earlier initiation of adjuvant TMZ CRT would be of clinical benefit. Up to half of glioblastoma patients experience radiological disease progression during the interval between surgery and adjuvant treatment with the tumor doubling time, the duration with which it grows to twice its previous volume, was documented to be as short as 24 days.^38–40^ This hypothesis was supported by a single institution study that noted a significant shortening of OS when CRT was initiated more than 6 weeks after surgery compared to within 2 weeks (aHR: 3.76; 95% CI: 1.01–14.57).^29^ These findings were subsequently corroborated by 2 additional studies that concluded that a delay in CRT by 6–9 weeks after surgery was associated with shorter OS.^22,30^

Counterintuitively, other investigators demonstrated a survival benefit for glioblastoma patients that had a moderate 4–6 weeks delay in the commencement of adjuvant therapy after surgery.^15,19^ A pooled analysis of 16 Radiation Therapy Oncology Group (RTOG) randomized trials from the pre-TMZ era revealed that deferring RT by more than 4 weeks provided a significant survival benefit compared to initiating RT within 2 weeks (12.5 versus 9.2 months, *P* < .0001).^41^ Studies in the TMZ era also observed improved OS among patients who started treatment at similar time intervals. A retrospective review of the National Cancer Database (NCDB) from 2004 to 2015 observed a modest increase in OS from 13.9 to 15.2 months when CRT was started at 4–6 weeks, compared to less than 4 weeks.^15^ This was supported by another retrospective study that identified early initiation of adjuvant TMZ CRT within 4 weeks was associated with poorer OS compared to 4–6 weeks (aHR: 1.31; 95% CI: 1.152–1.491).^19^ There are several possible reasons for  this phenomenon. First, early postoperative peritumoral hypoxia and edema were proposed to contribute to transient radioresistance.^42,43^ A moderate delay between surgery and CRT would allow for these postoperative changes in the tumor microenvironment to resolve. Such a delay would also permit radiation oncologists to better delineate gross tumor volumes as well as organs-at-risk more accurately during RT planning with updated imaging, mitigating the functionally detrimental adverse effects of radiation toxicity which has been identified to be an independent predictor for poorer OS.^44^ Finally, selection bias may explain why observed delays in treatment were beneficial. Patients with unfavorable prognostic characteristics, such as those that only received a tumor biopsy due to poor preoperative KPS, may have been subjected to earlier CRT.^45^

In the TMZ era, an increasing number of studies reported neither a beneficial nor harmful effect of the timing of postoperative CRT on survival outcomes.^21,46–49^ A pooled analysis of 1463 patients from 2 RTOG randomized trials did not detect an association between adjuvant therapy timing and OS.^11,27,50^ The inconclusive findings of these studies could be explained by their heterogeneous patient population that reviewed those that received either TMZ CRT or RT alone as a single cohort and had arbitrarily-defined S-CRT intervals, varying from 1 to 12 weeks after surgery.^15,16,20,21,48^ These studies also did not adjust for important prognostic factors, such as EOR or p*MGMT* methylation status.^15,16,20,21,48^ These issues were addressed in our study by including a relatively more homogeneous cohort of newly diagnosed glioblastoma, ie *IDH-1* wildtype WHO grade 4 astrocytomas, patients that only received adjuvant TMZ CRT. Confounding factors were also controlled when patient baseline characteristics were confirmed to be comparable between the various S-CRT interval groups. p*MGMT* methylation status is a well-recognized prognostic and predictive biomarker for glioblastoma.^7^ Furthermore, in light of the latest fifth WHO classification of central nervous system tumors, *IDH-1* mutant grade 4 astrocytomas are now recognized as a distinct diagnosis that carries a notably better prognosis, and had to be excluded from our analysis.^51^ Despite their significance, several studies that reviewed the timing of CRT failed to account for these important molecular markers, and with regard to p*MGMT* methylation, we continued to observe its independent influence on OS.^16,22,28^ Few studies included EOR in their analyses.^15,20,28,32,52^ One retrospective study of 138 patients, noted that among non-GTR patients, those initiating CRT within 4 weeks had significantly longer OS (11 verus 5 months).^32^ Another study of 161 patients discovered that among non-GTR patients, starting RT after 4 weeks improved OS from 7.8 to 12.3 months.^20^ Neither of these findings could be replicated in the current study that reviewed a considerably larger cohort of subjects. Only one study comprehensively analyzed the aforementioned conventional survival predictors for 209 glioblastoma patients and reached similar conclusions as ours indicating that the timing of CRT did not play a prognostic role.^53^

Several study limitations exist. First, EOR data was retrieved from either operative records or when available, early postoperative MRI scans.^54^ The major reason why we relied on such assessments was because of the absence of standard imaging protocols in Hong Kong where only 2 of the 7 neurosurgical centers offer routine early postoperative scanning. Second, a comparison of the S-CRT intervals of patients with rapid tumor recurrence before or during CRT with those who did not experience such swift disease progression would have been useful in defining a high-risk group that required earlier adjuvant therapy. Less than 5% of patients received CRT between 9 and 12 weeks, and such a small subgroup may not have been significantly powered to perform in-depth analysis. Third, the current study defined glioblastoma patients according to the fourth WHO classification. The latest fifth edition recently refined the diagnosis of glioblastoma by adopting a multilayered integrated approach incorporating new molecular criteria such as *TERT* promoter mutation, *EGFR* amplification, or chromosomal 7 gain/chromosomal 10 loss for *IDH-1* wildtype astrocytomas.^51^ Since the majority of lower grade astrocytomas in Hong Kong were not subject to such testing during the review period, a proportion of tumors would have been inadvertently excluded from the Hong Kong GBM Registry. Finally, the majority of registry patients (51%, 510/1006) only received adjuvant RT. According to an epidemiological study regarding patterns-of-care in the city, most GBM patients (59%, 594/1010) had a preoperative KPS of <80.^5^ This was likely the predominant reason why many were not considered suitable to undergo standard-of-care TMZ chemoradiotherapy. It was hypothesized that Hong Kong clinical oncologists were practicing according to the subject inclusion criteria of the original RCT that established this treatment that stipulated patients had to have an Eastern Cooperative Oncology Group (ECOG) performance status of 0–2 in order to be considered eligible.^55,56^ Another contributory factor could be that 20% of patients were older than 70 years which would have influenced the neuro-oncologist’s decision to refrain from administering chemoradiotherapy.^5^ Despite the encouraging results of administrating TMZ concurrently with short-course RT in improving OS for patients ≥65 years old, it is still standard practice in Hong Kong to forgo chemotherapy for elderly patients due to contrasting evidence.^57^ Two prospective trials and 1 RCT of elderly glioblastoma patients, defined as either >65 or >60 years, compared up-front TMZ alone versus RT alone, and concluded that chemotherapy for this relatively frail population was detrimental for those with p*MGMT*-unmethylated tumors and did not demonstrate improved OS.^58–60^ Another reason why a considerable proportion of patients were not administered TMZ was because the drug was only made freely available for p*MGMT*-unmethylated GBM patients in 2015.^5^

This is one of the largest studies to comprehensively review the impact of the timing of adjuvant TMZ CRT after glioblastoma resection on OS. This study showed that within a 3-month postoperative period, the timing of initiating adjuvant TMZ CRT does not significantly impact patient survival even after adjusting for important factors such as EOR, p*MGMT* methylation status, and KPS. In view of these results, there is sufficient clinical equipoise to conduct a prospective trial to determine the optimal timing of adjuvant chemoradiotherapy.

## Data Availability

The datasets used and/or analyzed during the current study are available from the corresponding author upon reasonable request.
